# Photoelectrode Characteristics of Partially Hydrolyzed Aluminum Phthalocyanine Chloride/Fullerene C_60_ Composite Nanoparticles Working in a Water Phase

**DOI:** 10.3390/molecules170910801

**Published:** 2012-09-10

**Authors:** Shuai Zhang, Toshiyuki Abe, Tomokazu Iyoda, Keiji Nagai

**Affiliations:** 1 Chemical Resources Laboratory, Division of Integrated Molecular Engineering, Tokyo Institute of Technology, Yokohama 226-8503, Japan; Email: shuaizhang@cczu.edu.cn (S.Z.); iyoda.t.aa@m.titech.ac.jp (T.I.); 2 Center for Low-Dimensional Materials, Micro-Nano Devices and System, Changzhou University, Changzhou 213164, Jiangsu, China; 3 Department of Frontier Materials Chemistry, Graduate School of Science and Technology, Hirosaki University, 3 Bunkyo-cho, Hirosaki, Aomori 036-8561, Japan; Email: tabe@cc.hirosaki-u.ac.jp

**Keywords:** organophotocatalyst, fullerene, phthalocyanine, photoelectrochemistry

## Abstract

Photoelectrochemical measurements were used to study the photoelectrode characteristics of composite nanoparticles composed of fullerene C_60_ and partially hydrolyzed aluminum phthalocyanine chloride (AlPc). In cyclic voltammetry measurements, the electrodes coated with the composite nanoparticles were found to have photoanodic [electron donor: 2-mercaptoethanol (ME)] and photocathodic (electron acceptor: O_2_) characteristics similar to those of the vapor-deposited p/n junction electrode. Their photoanodic features were further investigated with respect to the transient photocurrent response to light irradiation and the dependence on ME concentration (under potentiostatic conditions), from which it was noted that there was a decrease in the initial spiky photocathodic current and saturation of the steady-state photoanodic current at a higher ME concentration. Thus, the reaction kinetics was probably dominated by charge transport process. Moreover, external and internal quantum efficiency spectrum measurements indicated that the composite nanoparticles responded to the full spectrum of visible light (<880 nm) for both the photoanodic and photocathodic current. The present research will assist comprehension of photocatalytic behavior of the composite nanoparticles.

## 1. Introduction

Phthalocyanines (Pcs) constitute a remarkably versatile and robust class of compounds with diverse technological applications. For instance, metallophthalocyanines, in which the two hydrogen atoms of the central cavity can be replaced by more than 70 metals [1], have been used as efficient biomimetic catalysts for oxidation, reduction, and other reactions of organic compounds [2,3,4,5,6]. Besides, due to Pcs’ thermal and chemical stability, intense absorption in the red/near-infrared (IR) region of the solar spectrum, quite high fluorescence quantum yields, and capability to act as an electron donor for the acceptor moiety, they become valuable building blocks in organic photovoltaic devices [7,8,9,10,11,12,13], dye sensitized solar cells [14,15,16,17,18,19,20], photocatalysts [21,22,23,24,25,26,27,28], laser ablation control [29,30], and so on. In the application of photocatalysts, Pcs may act as photosensitizers to extend the absorption edge of some semiconductors with large band gap [21,22,23] through an intercomponent electron transfer. Otherwise, Abe and co-workers recently applied Pcs to organic photoelectrocatalysts with p/n junction and achieved water splitting under visible light and a small bias [24,25]. Nagai and co-workers combined the p/n layer with the absorbent Nafion and achieved photodegradation of trimethylamine to CO_2_ without bias [26]. Based on the report about the organophotocatalysts, we further developed nanoparticulate organic photocatalysts composed of C_60_ and partially hydrolyzed aluminum phthalocyanine chloride (AlPc) [27,28]. The composite exhibited photocatalytic superiority over single component nanoparticles for photodegradation of various organic substrates to CO_2_ without absorbent or bias, and its CO_2_ action spectrum for decomposition of 2-mercaptoethanol (ME) covered almost full spectrum of visible light [28]. However, the role of the composite in the photocatalysis was still unclear.

Photoelectrochemistry is one of the most promising methods for investigating and establishing photoenergy conversion systems, in which the photoelectrochemical water splitting system of a TiO_2_ electrode [31] as well as the dye-sensitized photovoltaic cell of a Ru complex absorbed on TiO_2_ electrode [32] are typical examples. It has also been used to study photo-induced oxidation and reduction reactions separately for the organic photoelectrocatalysts [24,25,33,34,35] or photocatalysts [26] with p/n junction. In the present research, the photoanodic and photocathodic characteristics of the ME oxidation and the reduction involving O_2_, respectively for the AlPc/C_60_ composite nanoparticles were investigated by the method. The research will offer benefit for comprehension of photocatalytic behavior of the composite nanoparticles.

## 2. Results and Discussion

### 2.1. Comparison of Photoanodic Characteristics for Electrodes Composed of Different Nanoparticles

**Figure 1:**
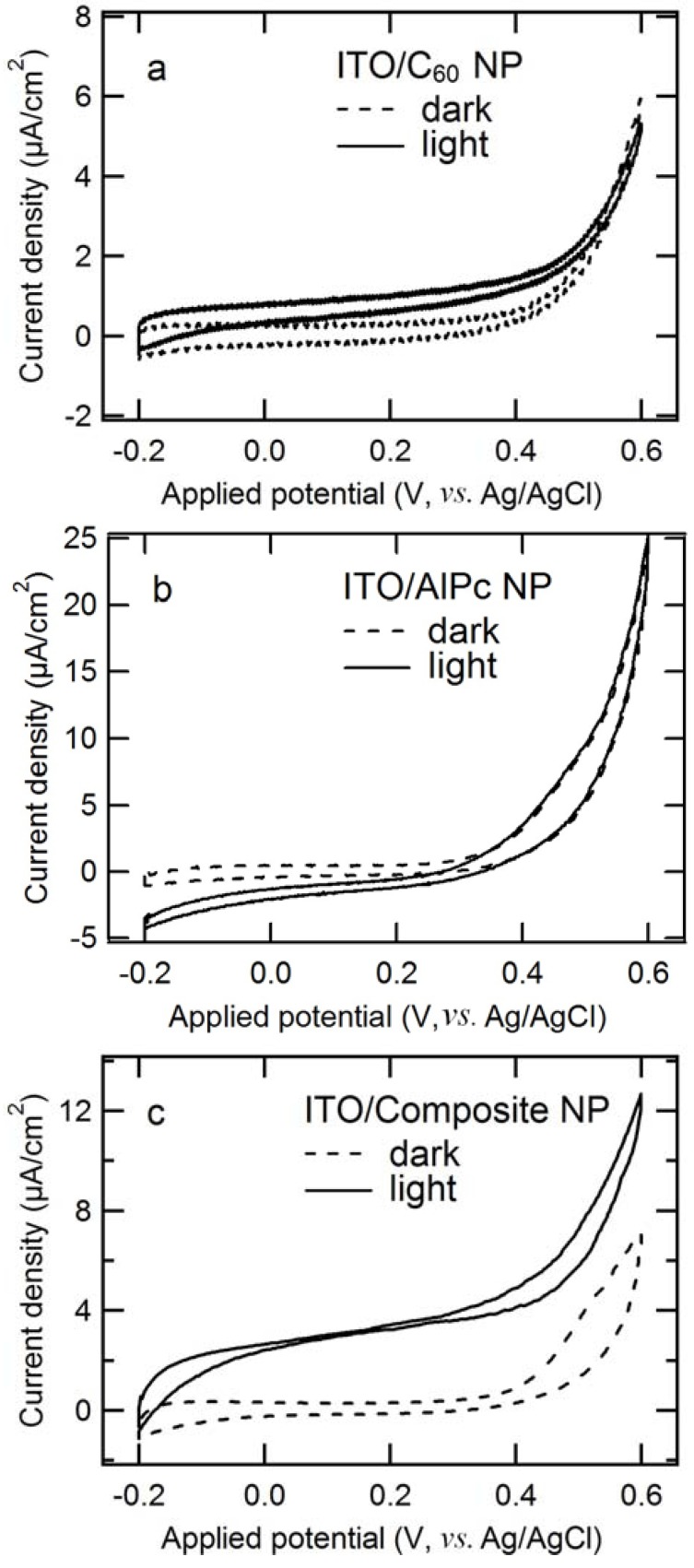
Cyclic voltammograms of particle films of (**a**) C_60_; (**b**) AlPc; and (**c**) composite on ITO: solid line, under illumination; dashed line, in the dark. The electrolyte solution: 2 × 10^−3^ mol/L 2-mercaptoethanol (PH ~ 10) in an Ar atmosphere; Scan rate: 20 mV/s; light intensity: 100 mW/cm^2^. The composite’s loading mole ratio of AlPcCl to C_60_ was 5:4.

The photoanodic characteristics of nanoparticle electrodes were first investigated by means of cyclic voltammetry. In our previous research [27], a comparison of the photoanodic threshold potential values between the nanoparticles and vapor-deposited films demonstrated that the composite nanoparticles have photoelectrochemical characteristics similar to the C_60_/AlPcCl layer, where trimethylamine (TMA) was used as the electron donor. In this study, the nanoparticle electrodes exhibit the same threshold potential values as those in the previous research: −0.2 V, ~+0.4 V (anodic current), and −0.2 V for the C_60_, AlPc, and composite nanoparticles, respectively (obtained from Figure 1), although the electron donor was changed from TMA to ME. Furthermore, single component nanoparticles exhibit a small net photoanodic current and only an anodic dark current for the C_60_ (Figure 1a) and AlPc nanoparticles (Figure 1b), respectively, as a result of the semiconductor/liquid interface of a Schottky junction [36]. The composite nanoparticles (Figure 1c) show a superior net photoanodic current as compared to that of either C_60_ or AlPc. The results demonstrate that the photoanodic characteristics of the nanoparticles are based on the semiconducting characters of the corresponding p- or n-type semiconductor layer for the single-component nanoparticles and the formation of p/n junction for the composite nanoparticles.

### 2.2. Kinetic Characteristics of Photoanode Composed of Composite Nanoparticles

#### 2.2.1. Transient Photoanodic Current Generated at ITO/Composite Nanaoparticles

**Figure 2:**
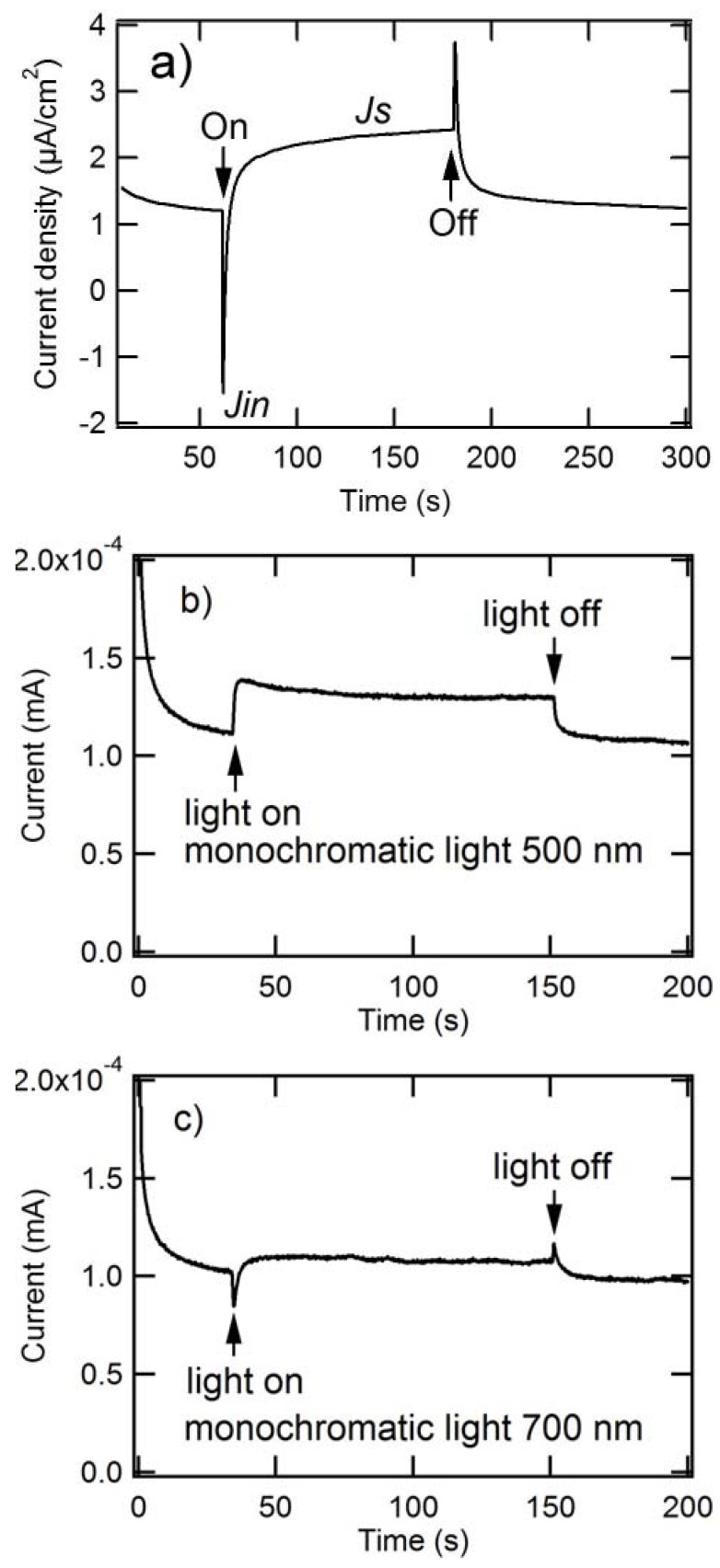
Time course of photocurrent generated by ITO/composite electrode: (**a**) The electrolyte contained 0.5 mmol/L 2-mercaptoethanol (PH ~ 10) in an Ar atmosphere; applied potential = +0.3 V (*vs*. Ag/AgCl); light intensity = 100 mW/cm^2^. (**b**) and (**c**) The electrolyte contained 2 mmol/L 2-mercaptoethanol (PH~10) in an Ar atmosphere; Applied potential = +0.3 V (*vs*. Ag/AgCl); the number of incident photon was adjusted to 3.5 × 10^13^ cm^−2^s^−1^ at different wavelength. The composite’s loading mole ratio of AlPcCl to C_60_ was 5:4.

**Figure 3:**
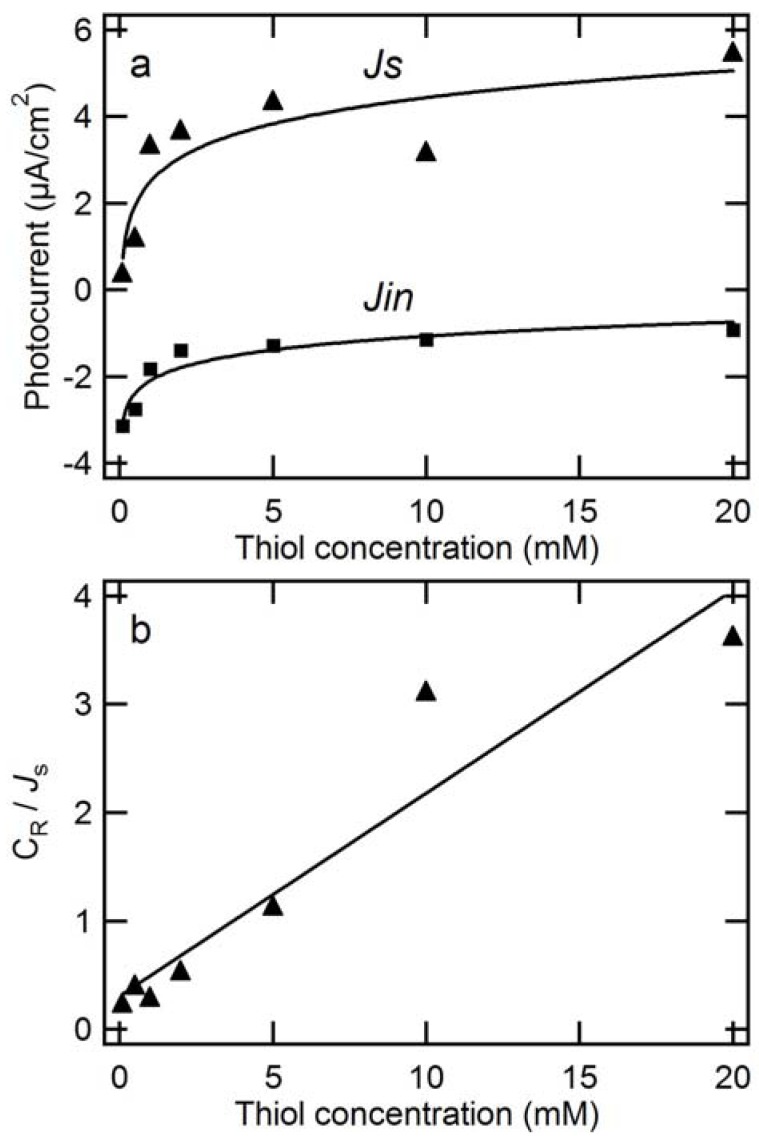
(**a**) The dependencies of the initial spiky photocurrent (*J*_in_, ■,) as well as steady-state photocurrent (*J*_s_, ▲,) on the ME concentration. (**b**) The plots of the ME concentration in the electrolyte (*C*_R_) / the steady-state photocurrent (*J*_s_) *vs*. *C*_R_. Applied potential = +0.3 V (*vs*. Ag/AgCl); light intensity = 100 mW/cm^2^; measured in an Ar atmosphere. The composite’s loading mole ratio of AlPcCl to C_60_ was 5:4.

To investigate the kinetic characteristics of ME oxidation at the composite nanoparticle/water interface, measurements of the photoelectrochemical response to light irradiation were carried out. Figure 2a shows a typical photoelectrochemical response of the electrode (ITO/composite nanoparticles) immediately after irradiating a white light (intensity: 100 mW/cm^2^) under potentiostatic conditions. Initially, a spiky photocurrent (*J*_in_) is observed, which then attains a steady-state (*J*_s_ represents the steady-state photocurrent). However, the sign of the photocurrent spike (cathodic) is the opposite of that of the steady-state anodic photocurrent, which is different from the reported electrode of ITO/C_60_/H2Pc [36]. The observed spiky photocathodic current is probably composed of two contributions: a photoanodic contribution due to ME oxidation [36] and a photocathodic contribution [37] due to several possible reasons (*vide post*). The former increases with the ME concentration, while the later remains constant, so the resultant photocathodic current decreases with the ME concentration (*vide post*, Figure 3a, *J*_in_). The origin of the photocathodic contribution is still unclear, but several factors are suggested. The first is the formation of O_2_-bridged metal Pc dimer [38], for which the photocathodic contribution may be the result of O_2_ reduction through electron transfer from photoexcited AlPc to oxygen. Second, it has been reported that Pcs may undergo photo-oxidation involving singlet oxygen addition to the macrocyclic rings [39], which can be achieved in the particle preparation process of the present research. The oxidized Pcs may be also reduced by electron transfer from photoexcited AlPc, and hence the photocathodic contribution is generated. These two hypotheses are consistent with the wavelength dependency of the photocathodic spike (Figure 2b,c): the unusual spike disappears at the wavelength (*i.e.*, 500 nm) where AlPc has little absorbance, whereas it appears at 700 nm where AlPc has remarkable absorbance, but they cannot account for the photoanodic spike observed immediately after blocking light (Figure 2a,c), which indicates the existence of reversible reactions. Further considering that the absolute value of the photocathodic spike is larger than that of the photoanodic one (Figure 2a,c), it is found that the reactions corresponding to the photocathodic spike are partially reversible. Therefore, the other two possibilities are also proposed: the rearrangement of the electrical double layer in response to photogenerated charge carriers and the presence of localized midgap states in AlPc. They were also considered as the reasons for the unusual photocathodic and photoanoic spikes for the nickel hydroxide thin-film electrode after irradiating and blocking light, respectively [37].

#### 2.2.2. Dependence of Photocurrent on ME Concentration

Figure 3a shows that the steady-state anodic photocurrent increases, whereas the spiky photocathodic one decreases with the ME concentration. Both the photocurrents deviate from the linear relationship (Figure 3a). If the steady-state anodic photocurrent increased linearly with the ME concentration, it would indicate that the electrode kinetics were completely dominated by a mass transport of ME (electron transport from ME to the electrode took place efficiently, and the mass transport process was the rate-determining step). However, in the present system, this is not the case. The results show that the photocurrent deviates from the linear relationship even in the low concentration region (*i.e.*, order of mmol/L), which probably indicates that the electrode reaction kinetics are dominated by an electrochemical process (*i.e.*, charge transport between ME and AlPc) [36]. The steady-state photocurrent (*J*_s_) may be analyzed to assume a Langmuir adsorption prior to the rate-limiting charge transfer step, where only the adsorbed ME with a surface concentration (*Γ*) is photoelectrochemically oxidized and the reaction products are quickly desorbed from the semiconductor surface [36,40]. Therefore the following two equations are valid:


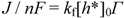
(1)


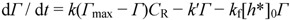
(2)

where *n*, the number of electrons transferred to a thiol molecular; *F*, Faraday’s constant; *k*_f_, rate constant of the electrochemical reaction; [h*]_0_, the surface concentration of the holes; *k*, the rate constant of adsorption; *k*’, the rate constant of desorption; *C*_R_, the thiol concentration in the electrolyte; and *Γ*_max_, the postulated maximum coverage when all available sites are occupied.

In the steady-state, from d*Γ* / d*t* = 0, Equation (3) is derived. Furthermore, Equation (4) is obtained by inserting *J* from Equation (1) into Equation (3):


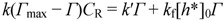
(3)


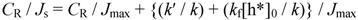
(4)

where *J*_max_ is the postulated photocurrent from *Γ*_max_. The plot of *C*_R_/*J*_s_ *vs*. *C*_R_ (Figure 3b) shows a linear relationship that is constant with Equation (4). From the slope of the line, *J*_max_ was calculated to be 6.3 μA/cm^2^. The value is 100 times smaller than that of the reported organic electrode [36] which reached 6.3 × 10^2^ μA/cm^2^, probably due to the poor electron injection into the ITO electrode and random orientations of the composite nanoparticles to the base electrode [27].

### 2.3. EQE and IQE Spectra for Steady-State Photoanodic Current

**Figure 4:**
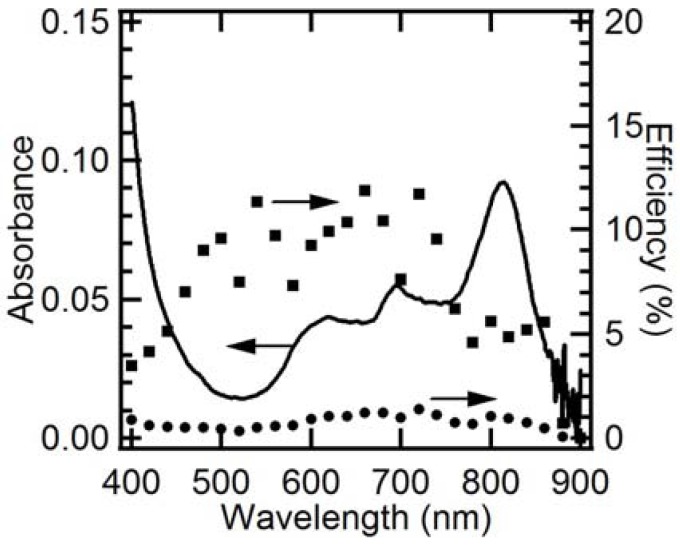
Steady-state EQE (●) and IQE (■) spectra for photoanodic current generated at ITO/composite nanoparticles and absorption spectrum of the electrode employed (solid line). The number of incident photon was adjusted to 3.5 × 10^13^ cm^−2^s^−1^ at each wavelength. ME concentration, 10 mM (pH, 10); Applied potential, +0.3 V; measured in an Ar atmosphere. The composite’s loading mole ratio of AlPcCl to C_60_ was 5:4.

**Figure 5:**
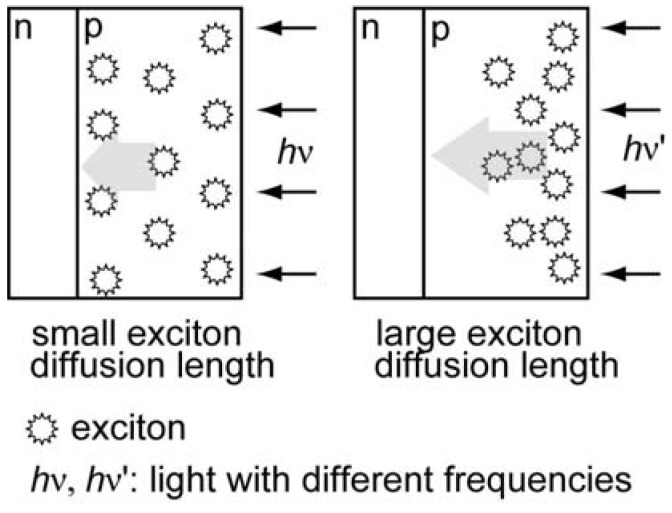
Schematic representation of the correlation between absorption coefficient and exciton diffusion length. The p-type semiconductor has large absorption coefficient at the frequency ν′, and small coefficient at the ν.

In order to determine the origin of the photoanodic current, its EQE and IQE spectra were measured (Figure 4). The resulting EQE spectrum covers the full spectrum of visible light, even reaching 860 nm, which is consistent with the composite’s absorption edge. Moreover, it almost corresponds with the absorption spectrum, which demonstrates that the present photocurrent generation is induced by the light absorption of both C_60_ and AlPc in the composite. A similar phenomenon was observed for the vapor-deposited organic electrode of C_60_/H_2_Pc [36,41]. The photoanodic current (Figure 1c) is mainly derived from the ME oxidation by holes in AlPc that are generated through the migration of the excitation energy of C_60_ and/or AlPc and the following charge separation at p/n junction, by taking into account the charge separation characteristics of the composite (because of its photocurrent superiority, as shown in Figure 1). The IQE values are absorbance-dependent: high absorbance mainly gives small IQE values, and low absorbance gives large IQE values. It can be interpreted according to an optical filter effect by AlPc itself and the higher probability of exciton diffusion to the p/n interface in the case of low absorbance [27] as shown in Figure 5. Although the IQE values are lower around the wavelength of 800 nm where AlPc has large absorbance, the dye still has important role for the composite: The AlPc material can form a heterojunction with C_60_ in the composite and enhance charge separation. Hence, the net photoanodic current (Figure 1) of the composite is superior to that of the single component C_60_ or AlPc. In addition, the presence of AlPc also increases light harvesting compared to the single component C_60_.

### 2.4. Comparison of Photocathodic Characteristics for Vapor-Deposited Electrodes

Photoinduced oxidation and reduction exist simultaneously in photocatalytic reactions. The former has been studied for composite nanoparticles in terms of the photoanodic characteristics (Figure 1, Figure 2, Figure 3, Figure 4). To further understand the composite nanoparticles’ photocatalytic mechanism, photoelectrochemical methods were also used to study their photocathodic properties relating to O_2_ reduction. First of all, as a reference for the nanoparticles, vapor-deposited organic semiconductor electrodes composed of C_60_ and/or AlPcCl were studied. Figure S1 shows the photocathodic currents for all the samples, and their threshold potential values are ~+0.14 V, ~+0.33 V, ~+0.33 V, and ~+0.4 V for ITO/C_60_, ITO/AlPcCl, ITO/C_60_/AlPcCl, and ITO/AlPcCl/C_60_, respectively. The photocathodic currents of the ITO/C_60_/AlPcCl and ITO/C_60_ electrodes showe saturation at potentials of about 0.3–0.0 V and 0.2–0.0 V, respectively, while the ITO/AlPcCl/C_60_ and ITO/AlPcCl electrodes showe notable photocathodic current increase at potentials of about 0.4–0.0 V and 0.3–0.0 V, respectively, which agrees with the scheme (Figure S2). The larger net photocathodic current for ITO/AlPcCl/C_60_ compared to that for ITO/C_60_ implies the efficient reduction involving oxygen by electrons in C_60_ generated at the AlPcCl/C_60_ interface.

### 2.5. Comparison of Photocathodic Characteristics for Electrodes Composed of Different Nanoparticles

**Figure 6:**
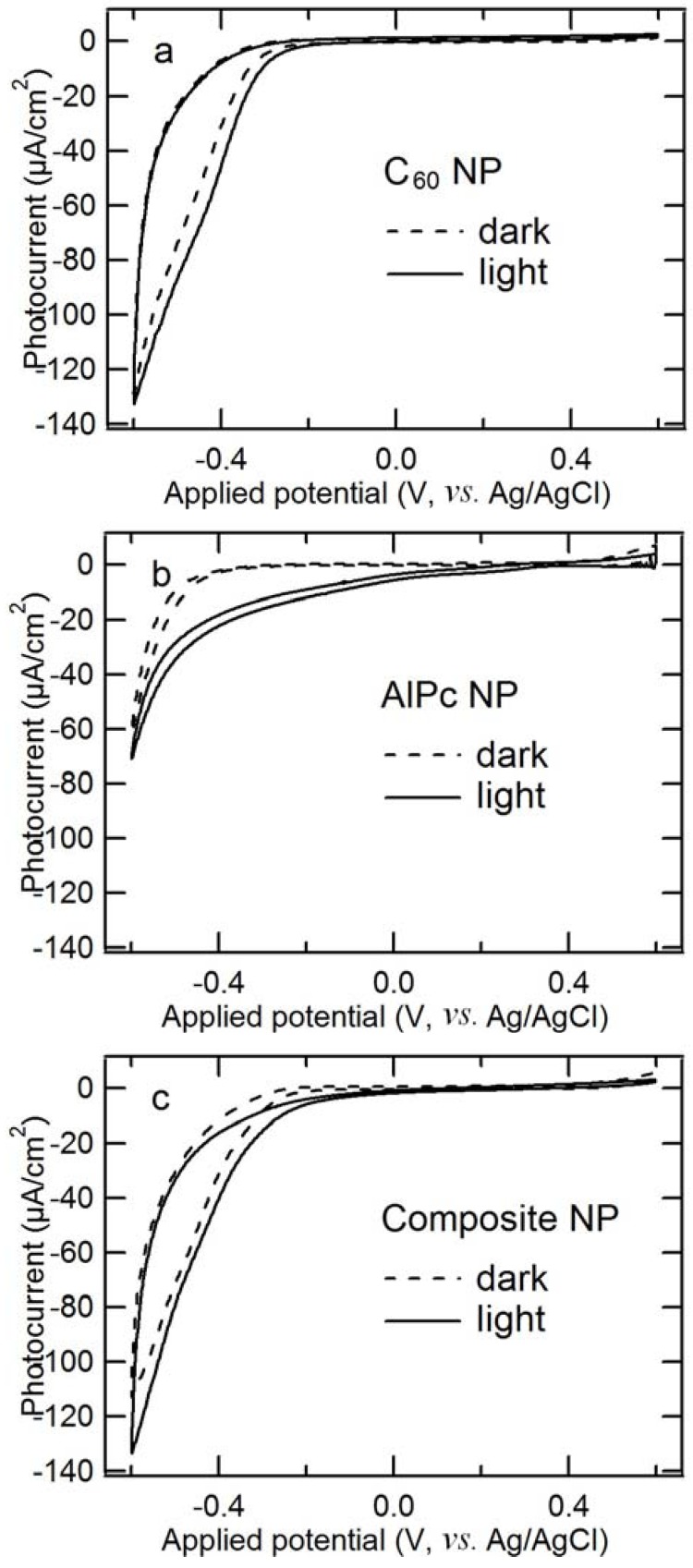
Cyclic voltammograms of particle films of (**a**) C_60_, (**b**) AlPc, and (**c**) composite on ITO: solid line, under illumination; dashed line, in the dark. The electrolyte solution contained 0.1 M KNO_3_ in an air atmosphere; Scan rate: 20 mV/s; light intensity: 100 mW/cm^2^. The composite’s loading mole ratio of AlPcCl to C_60_ was 5:4.

In the nanoparticle case, Figure 6 also shows the photocathodic currents for all of the samples. The photocathodic current is caused by the reduction involving oxygen because it almost disappears after Ar was bubbled into the electrolyte (Figure S3). The threshold potential values are ~+0.10 V, ~+0.33 V, and ~+0.33 V for the C_60_, AlPc, and composite nanoparticles, respectively. This shows the similarity between the nanoparticles and the corresponding vapor-deposited electrode (Figure S1), demonstrating that the photocathodic characteristics of the nanoparticles are also based on the semiconducting characteristics of the p, n, or p/n layer. The threshold potential value of the composite nanoparticles (Figure 6c) is a little less positive than that of the p/n layer (Figure S1d) as a result of random orientations of the composite nanoparticles relative to the ITO electrode surface [27]. The observed photocathodic current indicates that the consumption of photoexcited electrons in the composite may be through a multielectron reduction of O_2_ according to the conduction band level of C_60_ [42].

### 2.6. EQE and IQE Spectra for Steady-State Photocathodic Current

**Figure 7:**
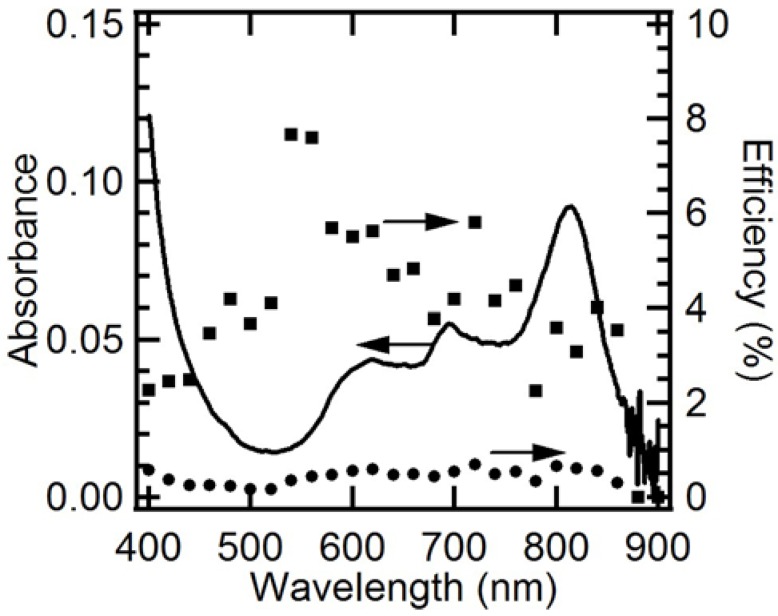
Steady-state EQE (●) and IQE (■) spectra for photocathodic current generated at ITO/composite nanoparticles and absorption spectrum of the electrode employed (solid line). The number of incident photon was adjusted to 3.5 × 10^13^ cm^−2^s^−1^ at each wavelength. The electrolyte contained 0.1 M KNO_3_ in an air atmosphere; Applied potential, +0.3 V. The composite’s loading mole ratio of AlPcCl to C_60_ was 5:4.

Figure 7 shows the EQE and IQE spectra of the photocathodic current involving oxygen for the composite particles. Similar to the spectra of the photoanodic current in Figure 4, it is found that the EQE spectrum in Figure 7 is almost consistent with the absorption spectrum and covers the full spectrum of visible light, indicating that the present photocathodic current is generated by the light absorption of both C_60_ and AlPc in the composite. The photocathodic current may be derived from the reduction involving O_2_ by electrons that are generated through the migration of the excitation energy of C_60_ and/or AlPc and the following charge separation at Schottky or p/n junction, by taking into account the photocathodic characteristics of the different nanoparticles (Figure 6). The highest IQE value reaches 8%, and the values are also absorbance-dependent as discussed in the part of photoanodic characteristics.

## 3. Experimental

### 3.1. Materials

Fullerene C_60_ (99%) was obtained from Tokyo Chemical Industry Co., Ltd. (TCI), and was used as received. Aluminum phthalocyanine chloride (AlPcCl, TCI) was purified twice by sublimation prior to its use. An indium tin oxide-coated (ITO-coated) glass plate (resistance: 8 Ω cm^−2^; transmittance: >85%; thickness: 174 nm) was commercially obtained from Asahi Glass Co., Ltd., and was washed using sonication in water containing detergent, pure water, acetone, and ethanol, in that sequence. The ITO plate was dried in air before use. All the other solvents and chemicals were of reagent-grade quality, purchased commercially, and used without further purification.

### 3.2. Particle Synthesis and Electrode Preparation

Organic semiconductor nanoparticles composed of AlPc and/or C_60_ were prepared according to our previous research [27,28]. The loading mole ratio of AlPcCl to C_60_ was controlled to be 5:4. Working electrodes were prepared by simply casting particle suspensions after filtration on an ITO glass surface and drying at room temperature. For instance, 0.15 mL of 0.1 mg/mL suspension was cast on an ITO electrode with an effective area of 1 cm^2^.

### 3.3. Photoelectrochemical Measurements

All the photoelectrochemical measurements were carried out in a standard three-electrode system, which was equipped with a modified ITO working electrode, a Pt wire counter electrode, and a Ag/AgCl (in saturated KCl electrolyte) reference electrode, using an electrochemical workstation (Hokuto Denko HSV-100). The working electrode was placed in contact with different electrolytes in an Ar or air atmosphere. A halogen lamp was used as the light source, and light was illuminated on the working electrode from the ITO side. The light intensity of 100 mW/cm^2^ was monitored and corrected using an optical power meter (3A-SH, Ophir, Ltd.).

The external quantum efficiency (EQE) and internal quantum efficiency (IQE) spectra for the photoanodic and photocathodic current were measured using a halogen lamp (150 W) as the light source in combination with a monochromator (SG-100, Kohken Co., Ltd.). The incident photon number intensity was adjusted to 3.5 × 10^13^ cm^−2^s^−1^ at each wavelength and was monitored using the power meter. In the experiment, an electrolyte containing 1 × 10^−2^ mol/L 2-mercaptoethanol (ME) (pH ~ 10) in an Ar atmosphere or 0.1 mol/L KNO3 in an air atmosphere was used. The EQE and IQE were calculated using Equations (1) and (2), respectively:


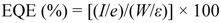
(5)


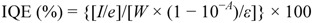
(6)

where *I* (A/cm^2^) is the photocurrent density; *e* (C) is the elementary electric charge; *W* (W/cm^2^) is the light intensity; *ε* is the photon energy (J); and *A* is the absorbance of the composite nanoparticles on the electrode.

## 4. Conclusions

We have investigated the photoelectrode characteristics of a nanoparticulate organic semiconductor composite. The electrode coated with the composite nanoparticles was found to carry out photo-induced oxidation of ME or reduction involving O_2_, and p/n junction of the composite nanoparticles further enhances the photoanodic current. EQE and IQE spectra of the electrode covers full spectrum of visible light (<880 nm) for the steady-state photoanodic and photocathodic current under potentiostatic conditions. The results reported herein may benefit comprehension of the photocatalytic behavior of composite nanoparticles.

## Supplementary Materials

Supplementary materials can be accessed at: http://www.mdpi.com/1420-3049/17/9/10801/s1.

## Notes

*Sample Availability*: Samples of the nanoparticles are available from the authors based on the Material Transfer Agreement with Tokyo Institute of Technology (http://ime.res.titech.ac.jp/syusyoku.html).
