# Supplementary material for: Photoelectrode Characteristics of Partially Hydrolyzed Aluminum Phthalocyanine Chloride/Fullerene C60 Composite Nanoparticles Working in a Water Phase
Source: Molecules. 2012 Sep 10;17(9):10801–15. doi: 10.3390/molecules170910801 (PMC6268333; doi:10.3390/molecules170910801)

Supporting Information

Figure S1. Cyclic voltammpgrams of vapor deposited films on ITO. The electrolyte contained 0.1 M KNO_3 in an air atmosphere; light intensity = 100 mW/cm^2; scan rate is 20 mV/s.

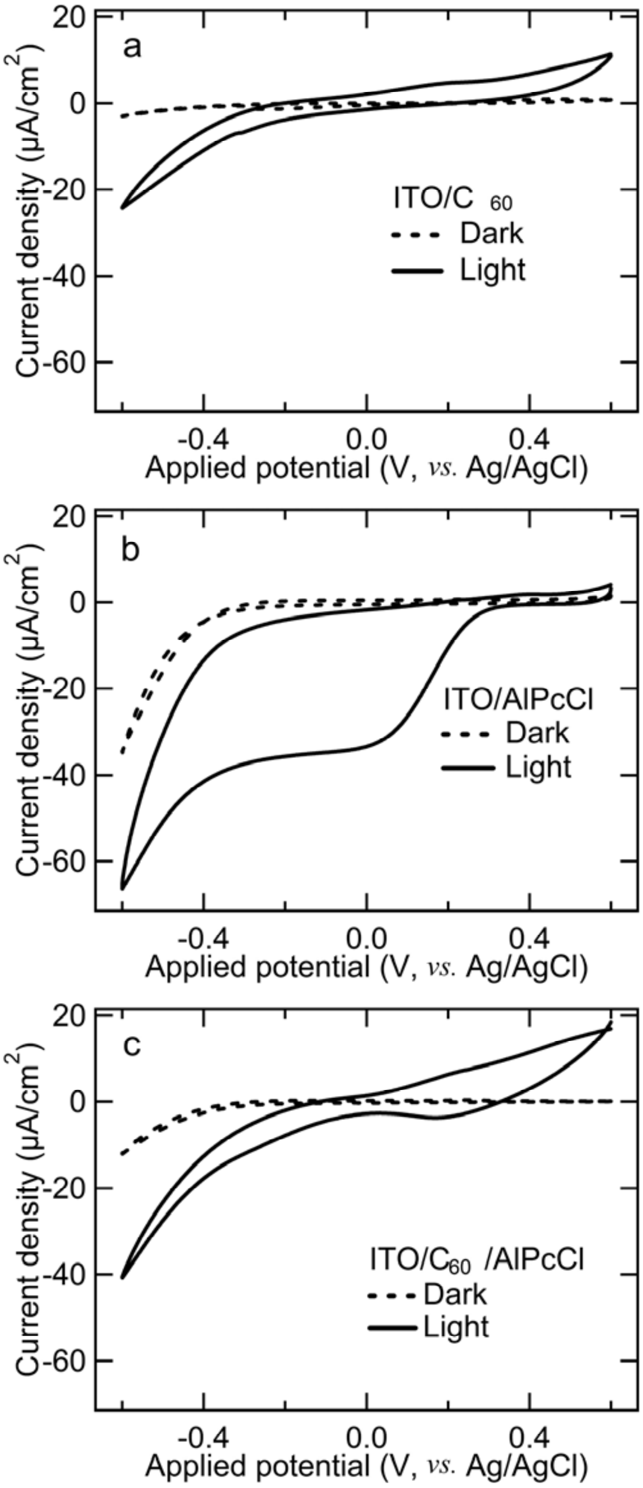

Figure S1. *Cont.*

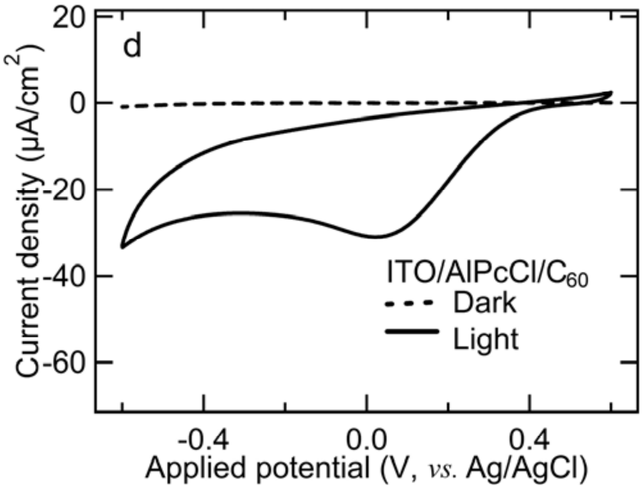

Figure S2. Schematic diagram of the photoelectrochemical processes for (**a**) an n-type monolayer; (**b**) a p-type monolayer; (**c**) an n/p bilayer; and (**d**) a p/n bilayer in the presence of an electron acceptor.

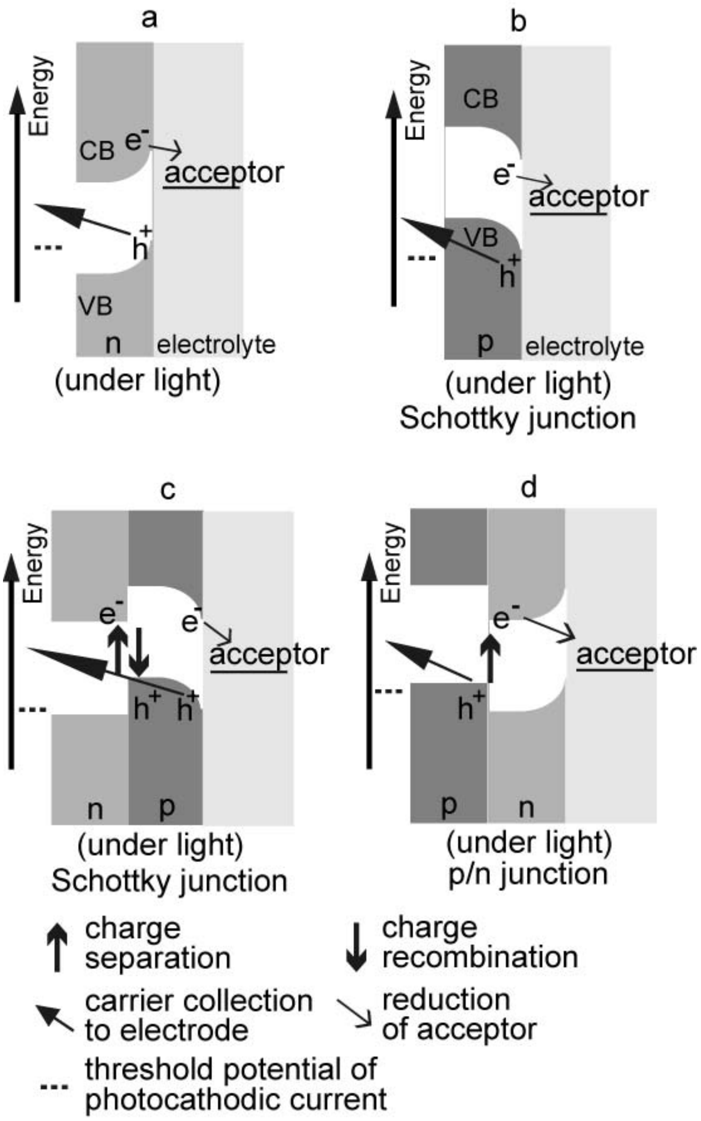

Figure S3. Cyclic voltammpgrams of nanoparticle films on ITO. The electrolyte contained 0.1 M KNO_3 in an Air (red lines) or Ar (dark lines) atmosphere; light intensity = 100 mW/cm^2; scan rate is 20 mV/s.

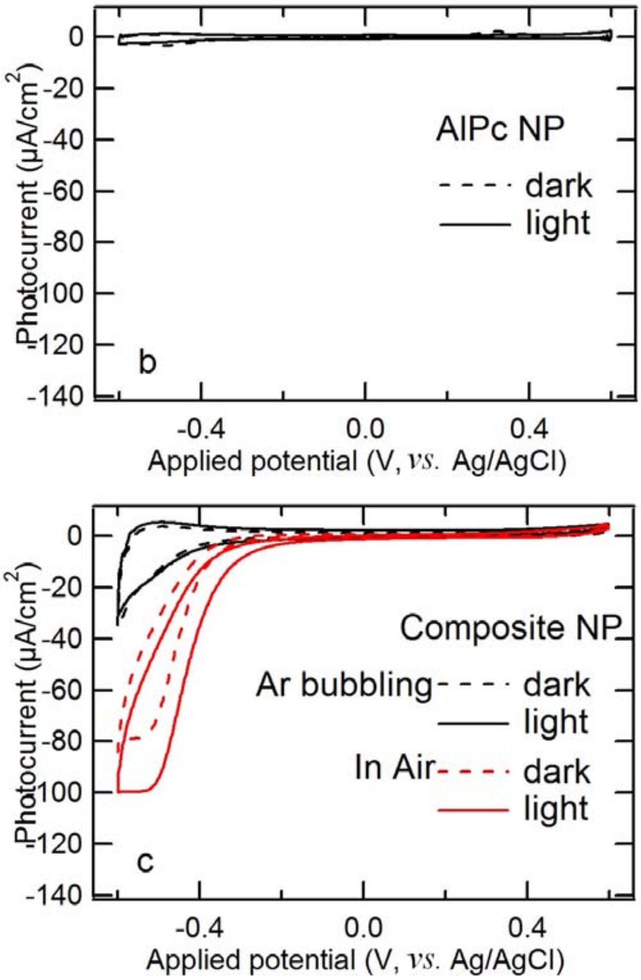

Supplement: Supplementary file 1 [file molecules-17-10801-s001.pdf]
